# Evaluating Bard Gemini Pro and GPT-4 Vision Against Student Performance in Medical Visual Question Answering: Comparative Case Study

**DOI:** 10.2196/57592

**Published:** 2024-12-17

**Authors:** Jonas Roos, Ron Martin, Robert Kaczmarczyk

**Affiliations:** 1 Department of Orthopedics and Trauma Surgery, University Hospital of Bonn, Venusberg-Campus 1, 53127, Bonn, Germany, 49 228-287-14170; 2 Department of Plastic and Hand Surgery, Burn Center, BG Clinic Bergmannstrost, Halle (Saale), Germany; 3 Department of Dermatology and Allergy, Technical University of Munich, Munich, Germany

**Keywords:** medical education, visual question answering, image analysis, large language model, LLM, student, performance, comparative, case study, artificial intelligence, AI, ChatGPT, effectiveness, diagnostic, training, accuracy, utility, image-based, question, image, AMBOSS, English, German, question and answer, Python, AI in health care, health care

## Abstract

**Background:**

The rapid development of large language models (LLMs) such as OpenAI’s ChatGPT has significantly impacted medical research and education. These models have shown potential in fields ranging from radiological imaging interpretation to medical licensing examination assistance. Recently, LLMs have been enhanced with image recognition capabilities.

**Objective:**

This study aims to critically examine the effectiveness of these LLMs in medical diagnostics and training by assessing their accuracy and utility in answering image-based questions from medical licensing examinations.

**Methods:**

This study analyzed 1070 image-based multiple-choice questions from the AMBOSS learning platform, divided into 605 in English and 465 in German. Customized prompts in both languages directed the models to interpret medical images and provide the most likely diagnosis. Student performance data were obtained from AMBOSS, including metrics such as the “student passed mean” and “majority vote.” Statistical analysis was conducted using Python (Python Software Foundation), with key libraries for data manipulation and visualization.

**Results:**

GPT-4 1106 Vision Preview (OpenAI) outperformed Bard Gemini Pro (Google), correctly answering 56.9% (609/1070) of questions compared to Bard’s 44.6% (477/1070), a statistically significant difference (*χ*^2^₁=32.1, *P*<.001). However, GPT-4 1106 left 16.1% (172/1070) of questions unanswered, significantly higher than Bard’s 4.1% (44/1070; *χ*^2^₁=83.1, *P*<.001). When considering only answered questions, GPT-4 1106’s accuracy increased to 67.8% (609/898), surpassing both Bard (477/1026, 46.5%; *χ*^2^₁=87.7, *P*<.001) and the student passed mean of 63% (674/1070, SE 1.48%; *χ*^2^₁=4.8, *P*=.03). Language-specific analysis revealed both models performed better in German than English, with GPT-4 1106 showing greater accuracy in German (282/465, 60.65% vs 327/605, 54.1%; *χ*^2^₁=4.4, *P*=.04) and Bard Gemini Pro exhibiting a similar trend (255/465, 54.8% vs 222/605, 36.7%; *χ*^2^₁=34.3, *P*<.001). The student majority vote achieved an overall accuracy of 94.5% (1011/1070), significantly outperforming both artificial intelligence models (GPT-4 1106: *χ*^2^₁=408.5, *P*<.001; Bard Gemini Pro: *χ*^2^₁=626.6, *P*<.001).

**Conclusions:**

Our study shows that GPT-4 1106 Vision Preview and Bard Gemini Pro have potential in medical visual question-answering tasks and to serve as a support for students. However, their performance varies depending on the language used, with a preference for German. They also have limitations in responding to non-English content. The accuracy rates, particularly when compared to student responses, highlight the potential of these models in medical education, yet the need for further optimization and understanding of their limitations in diverse linguistic contexts remains critical.

## Introduction

Large language models (LLMs) have gained attention in medical research and education, with an increasing recognition of their potential applications [1]. Recent literature highlights the outstanding capabilities of these LLMs, notably exemplified by OpenAI’s ChatGPT [2,3]. The introduction of the image recognition feature further expands the horizon, opening up a new realm of applications in medical clinical practice and research [4]. Previous studies on LLMs have demonstrated their ability to pass medical licensing examinations [5-7]. However, these studies were often limited by the models’ restricted image analysis capabilities, leaving some questions unanswered [7]. The detection of findings in a broad spectrum of medical fields, such as interpreting radiological imaging, identifying skin lesions in dermatology, analyzing electrocardiograms in cardiology, and understanding instrumental diagnostics such as sonography across various specialties, is particularly crucial. With the growing dependence on diagnostic imaging, highlighted by a notable increase in imaging procedures in hospitals, the ability to understand and interpret these images is becoming increasingly important [8]. Additionally, artificial intelligence (AI) presents a valuable opportunity to enhance the training and learning experience of medical trainees [9]. Therefore, it is essential for the future students to learn the terminology and fundamentals of AI, as well as receive training in the practical and critical application of algorithms, coupled with the development of reflective skills necessary in this evolving field [10].

In addition to ChatGPT’s latest version, GPT-4V, which excels in processing media content, the field of multimodal models is rapidly evolving [11]. Google Bard, launched on March 21, 2023, is designed as a counterpart to ChatGPT. Trained with similar data, Bard pursues objectives parallel to those of ChatGPT [12]. Since May 10, 2023, Bard has also been equipped with an image analysis feature [13].

Gemini Pro is an advanced AI model developed by Google DeepMind, known for its remarkable performance across a wide range of tasks, including image, audio, and video understanding [14]. The Gemini suite includes three sizes: Ultra, Pro, and Nano. The Pro version is designed to scale effectively across various tasks, making it versatile for different applications [15].

Gemini Pro has been integrated into Google’s AI chatbot Bard, resulting in significant improvement. Bard now has advanced capabilities in English language understanding, including advanced comprehension, planning, and task processing. It can respond to various types of inputs such as text, images, audio, video, and code [16].

We expect GPT-4V to outperform Gemini Pro in answering medical visual questions due to its advanced image processing capabilities. However, performance is expected to vary depending on the language of the questions. This study aims to investigate the potential and limitations of advanced LLMs with image recognition in medical education and diagnostics by comparing GPT-4V and Gemini Pro. Both models are expected to perform better on English questions than on German questions due to their training preferences and the complexity of medical terminology in different languages. The aim of this study is to evaluate and compare the effectiveness of GPT-4V and Gemini Pro in answering medical visual questions, especially on image-based multiple-choice questions from medical licensing examinations, to demonstrate their strengths and limitations and provide a basis for future improvements in medical education and practice.

## Methods

### Image-Based Multiple-Choice Questions

Questions were extracted from the learning platform “AMBOSS” based on specific criteria. AMBOSS is a comprehensive medical knowledge platform, available as a web-based tool and mobile app, designed to support medical students and professionals with constantly updated medical information and interactive tools [17]. Founded in 2012 in Berlin, Germany, AMBOSS offers various subscription plans starting around €11,99 per month (a currency exchange rate of US $1 = €0.9127 is applicable) for students and ranging from €16,50 to €22 per month for professionals [18].

The English-language version has also been available since 2018 [19]. AMBOSS is used by approximately 100,000 medical students preparing for their final medical licensing examinations, such as the Staatsexamen in Germany and the United States Medical Licensing Examination in the United States. These students leverage AMBOSS to review and test their knowledge across a broad range of medical topics [20].

These criteria included: questions that were not marked as outdated, containing only one image, and not being part of a multiple questions case series.

In total, we included 1070 questions using a standardized prompt for both models (Table 1) in the analysis, of which 605 were in English and 465 questions were in German.

Standardized prompts used for medical visual question-answering tasks in English and German. This table presents the system prompts provided to GPT-4 1106 Vision Preview and Bard Gemini Pro for answering 1070 image-based multiple-choice questions (605 English and 465 German) from the AMBOSS learning platform. The standardized format ensures consistency in the models’ approach to interpreting medical images and providing diagnoses or answers across both languages.

**Table 1.** Question template with language and prompt.

Language	Prompt
English	SYSTEM: Act as an expert physician and professor at a renowned university hospital. Your task is to answer medical questions, primarily based on descriptions of medical images. Use your expertise to interpret these descriptions accurately and provide the most likely diagnosis or answer.<QUESTION > <MULTIPLE-CHOICE-ANSWERS>Provide the answer to the multiple choice question in the format:<correct_letter>)<correct_answer>. Include a brief explanation if possible to support the answer.
German	SYSTEM: Stell dir vor, du bist ein erfahrener Arzt und Professor an einem renommierten Universitätskrankenhaus. Deine Aufgabe besteht darin, medizinische Fragen zu beantworten, die, sich vorwiegend auf Beschreibungen medizinischer Bilder stützen. Nutze deine Expertise, um diese Beschreibungen genau zu interpretieren und die, wahrscheinlichste Diagnose oder Antwort zu geben.<QUESTION><MULTIPLE-CHOICE-ANSWERS>Antworteauf die, Multiple-Choice-Frage im folgenden Format:<richtiger_Buchstabe>)<richtige_Antwort>. Gib wenn möglich eine kurze Erklärung zur Unterstützung deiner Antwort.

### Student Response

The analysis of student performance on various questions was based on response statistics from the AMBOSS platform. This platform provides the percentage of users who selected each possible answer for a given question, incorporating all responses recorded up to the reference dates (March 21, 2023, for German questions and June 16, 2023, for English questions).

The student passed mean represents the percentage of questions where the correct answer received a confidence rating above 60% from the students. For each question, we identified the correct answer among the student responses and checked if the confidence rate (percentage of students choosing this answer) for the correct answer was above 60% (eg, A: 64%, B: 5%, C: 4%, D: 2%, and E: 25%, with A being the correct answer). If the rate was above 60%, the question was considered “correct”; otherwise, it was considered “failed.” We then calculated the mean of these values across all questions.

The student majority vote determines whether the majority of students selected the correct answer for each question, serving as a gauge of collective consensus on the correctness of responses. If the majority of students chose the correct answer (eg, A: 25%, B: 20%, C: 20%, D:2 0%, and E: 15%, with A being the correct answer), the question was considered correctly answered by the majority; otherwise, it was not.

### Statistical Analysis

The analysis was conducted on an Apple M1 Pro macOS (version 14.2.1) system, using Python (version 3.10.12). We used several Python libraries for data analysis and visualization: Pandas (version 1.5.3) for data manipulation, Seaborn (version 0.11.2), and Matplotlib (version 3.7.2) for generating insightful plots, and Statannotations (version 0.6.0) to indicate statistical significance in our visual representations. The chi-square test was conducted to compare the accuracy of students and models, both overall and within languages. Additionally, we analyzed the feedback categories provided by Bard Gemini Pro, which included sexually explicit content, hate speech, harassment, and dangerous content.

### Declaration of Generative AI and AI-Assisted Technologies in the Writing Process

Grammarly and GPT-4 were used for language improvements and general paper revision. After using these tools, the authors reviewed and edited the content as needed and take full responsibility for the publication’s content.

### Ethical Considerations

The main goal of this study was to evaluate AI systems without having human volunteers directly involved. The potential impact of AI-produced medical content on clinical practice made accuracy the primary objective. The AI models produced content that was only used for study.

## Results

### Response Rate

**Figure 1.**
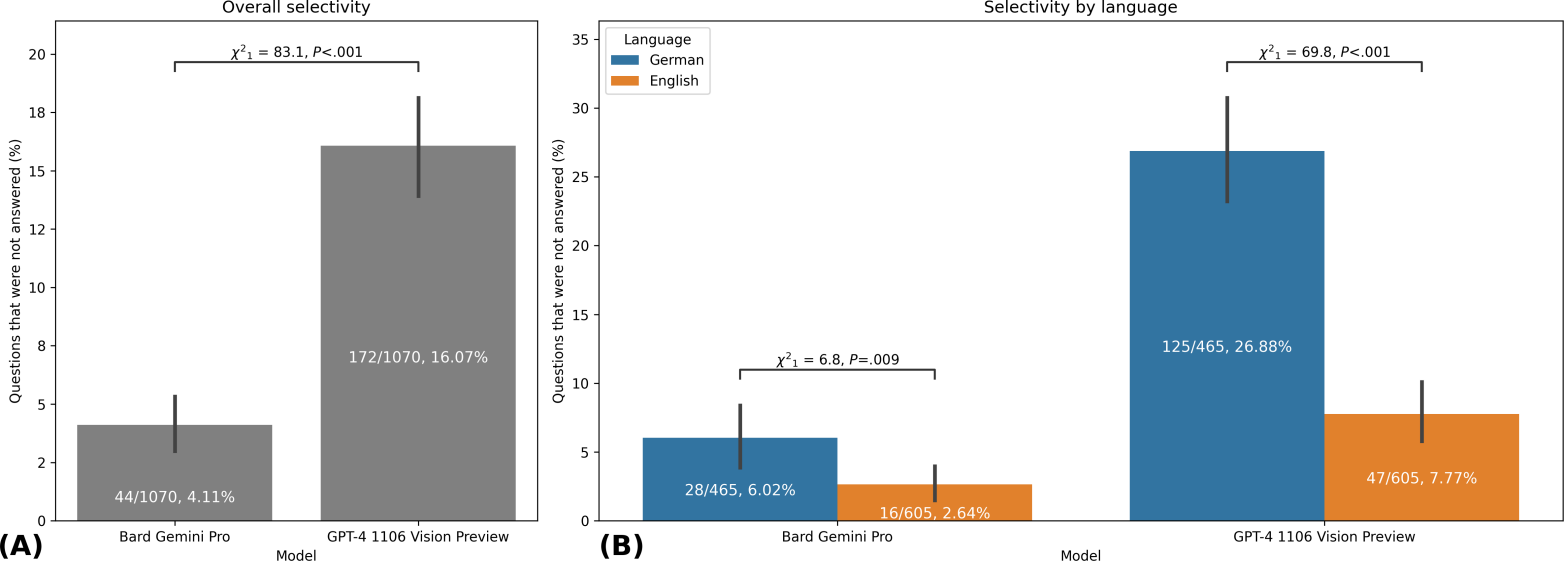
Selectivity analysis of AI models in answering medical visual questions across languages. This figure compares the proportion of unanswered questions by Bard Gemini Pro and GPT-4 1106 Vision Preview in a study involving 1070 image-based multiple-choice questions from the AMBOSS learning platform. (**A**) Overall selectivity: comparison of unanswered questions between models, showing GPT-4 1106 Vision Preview (172/1070, 16.07%) was significantly more selective than Bard Gemini Pro (44/1070, 4.11%; *χ*^2^₁=83.1, *P*<.001). (**B**) Selectivity by language: Both models showed higher selectivity for German questions compared to English. Bard Gemini Pro: German (28/465, 6.02%) versus English (16/605, 2.64%; *χ*^2^₁=6.8, *P*=.009). GPT-4 1106 Vision Preview: German (125/465, 26.88%) versus English (47/605, 7.77%; *χ*^2^₁=69.8, *P*<.001). This study was conducted in 2023, comparing AI model performance against medical student performance data from March 21, 2023 (German questions) and June 16, 2023 (English questions). The chi-square test was used for all statistical comparisons. Error bars represent 95% CIs of the mean. AI: artificial intelligence.

The GPT-4 1106 Vision Preview left a significantly larger number of questions unanswered compared to Bard Gemini Pro. Specifically, 16.1% (172/1070) of the questions remained unanswered for GPT-4 1106 Vision Preview, while Bard Gemini Pro left only 4.1% (44/1070) unanswered (*χ*^2^₁=83.1, *P*<.001). Interestingly, both the Bard Gemini Pro (*χ*^2^₁=6.8, *P*=.009) and the GPT-4 1106 Vision Preview (*χ*^2^₁=69.8, *P*<.001) were more selective when answering German questions (Figure 1).

### Overall Model Comparison

In terms of overall correctness of responses to medical visual question answering tasks, GPT-4 1106 Vision Preview outperformed Bard Gemini Pro. Specifically, GPT-4 1106 Vision Preview correctly answered 56.9% (609/1070) of the questions, whereas Bard Gemini Pro achieved a correct response rate of 44.6% (477/1070; *χ*^2^₁=32.1, *P*<.001). Students performed better with a mean correct answer rate of 63% (674/1070; *χ*^2^₁=8.0, *P*=.005 compared to GPT-4 1106 Vision Preview).

Moreover, when only considering the questions that were answered by each model, the performance gap between the two became even more apparent. GPT-4 1106 Vision Preview had a correct answer rate of 67.8% (609/898) for the answered questions, while Bard Gemini Pro had a correct answer rate of 46.5% (477/1026; *χ*^2^₁=87.7, *P*<.001). In this scenario, the GPT-4 1106 Vision Preview now surpasses the student passed mean (*χ*^2^₁=4.8, *P*=.03).

**Figure 2.**
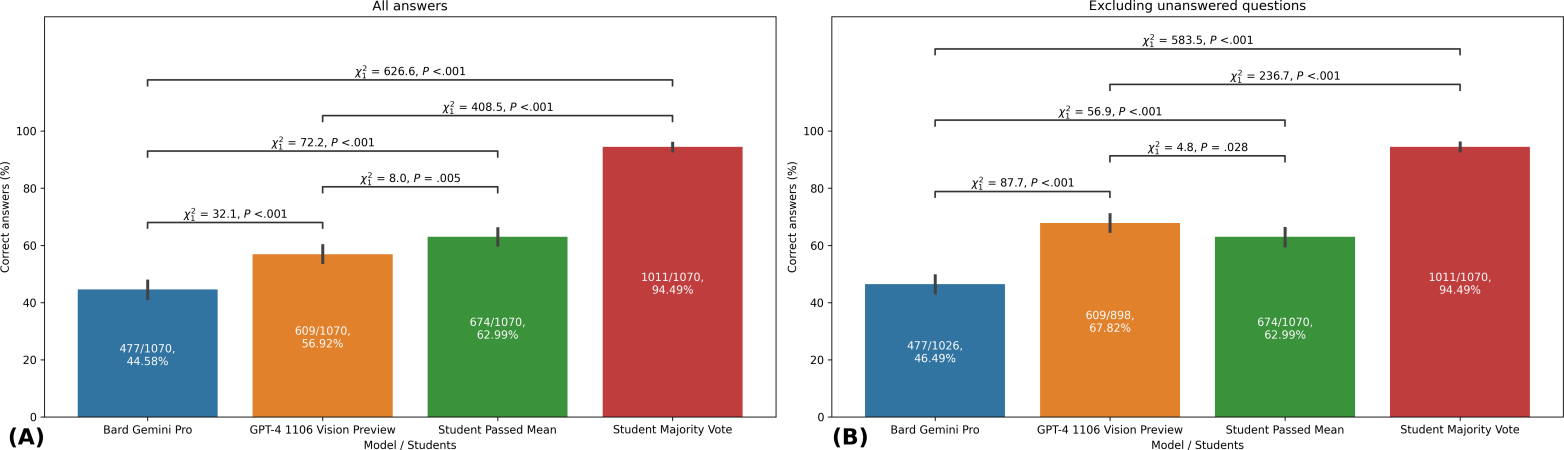
Comparative accuracy of AI models and medical students in answering image-based multiple-choice questions. This figure shows the overall accuracy for Bard Gemini Pro, GPT-4 1106 Vision Preview, student passed mean, and student majority vote in a medical visual question-answering task. This study analyzed 1070 image-based multiple-choice questions (605 in English and 465 in German) from the AMBOSS learning platform, covering various medical specialties. (**A**) Accuracy rates for all questions, including unanswered ones. (**B**) Accuracy rates excluding unanswered questions. The chi-squared test was used to compare accuracy across models and students. This study was conducted in 2023, comparing AI model performance against medical student performance data from March 21, 2023 (German questions) and June 16, 2023 (English questions). Error bars represent 95% CIs of the mean. Sample sizes (n) are provided for each group. AI: artificial intelligence.

The student collective majority vote revealed 94.5% (1011/1070) correctly answered questions, surpassing all other models and the student passed mean (GPT-4 1106 Vision Preview vs student majority vote: *χ*^2^₁=408.5, *P*<.001; Bard Gemini Pro vs student majority vote: *χ*^2^₁=626.6, *P*<.001, Figure 2).

### Model Comparison by Language

In the second part of our study, we compared the performance of Bard Gemini Pro and GPT-4 1106 Vision Preview, as well as the student passed mean and majority vote, in answering medical visual question answering tasks in two different languages: German and English. Our analysis revealed significant differences in the performance of these models based on the language of the questions.

Specifically, GPT-4 1106 Vision Preview had better accuracy in German (282/465, 60.65%) compared to English (327/605, 54.1%; *χ*^2^₁=4.4, *P*=.04). The trend was more pronounced when only considering answered questions (German: 282/340, 82.9%, English: 327/558, 58.6%; *χ*^2^₁=56.2, *P*<.001).

Bard Gemini Pro also revealed significant performance variations, with a higher accuracy in German (255/465, 54.8%) than in English (222/605, 36.7%; *χ*^2^₁=34.3, *P*<.001). This pattern persisted across all answered questions, indicating a consistent language-based performance gap (German: 255/437, 58.4%; English: 222/589, 37.7%; *χ*^2^₁=42.2, *P*<.001).

**Figure 3.**
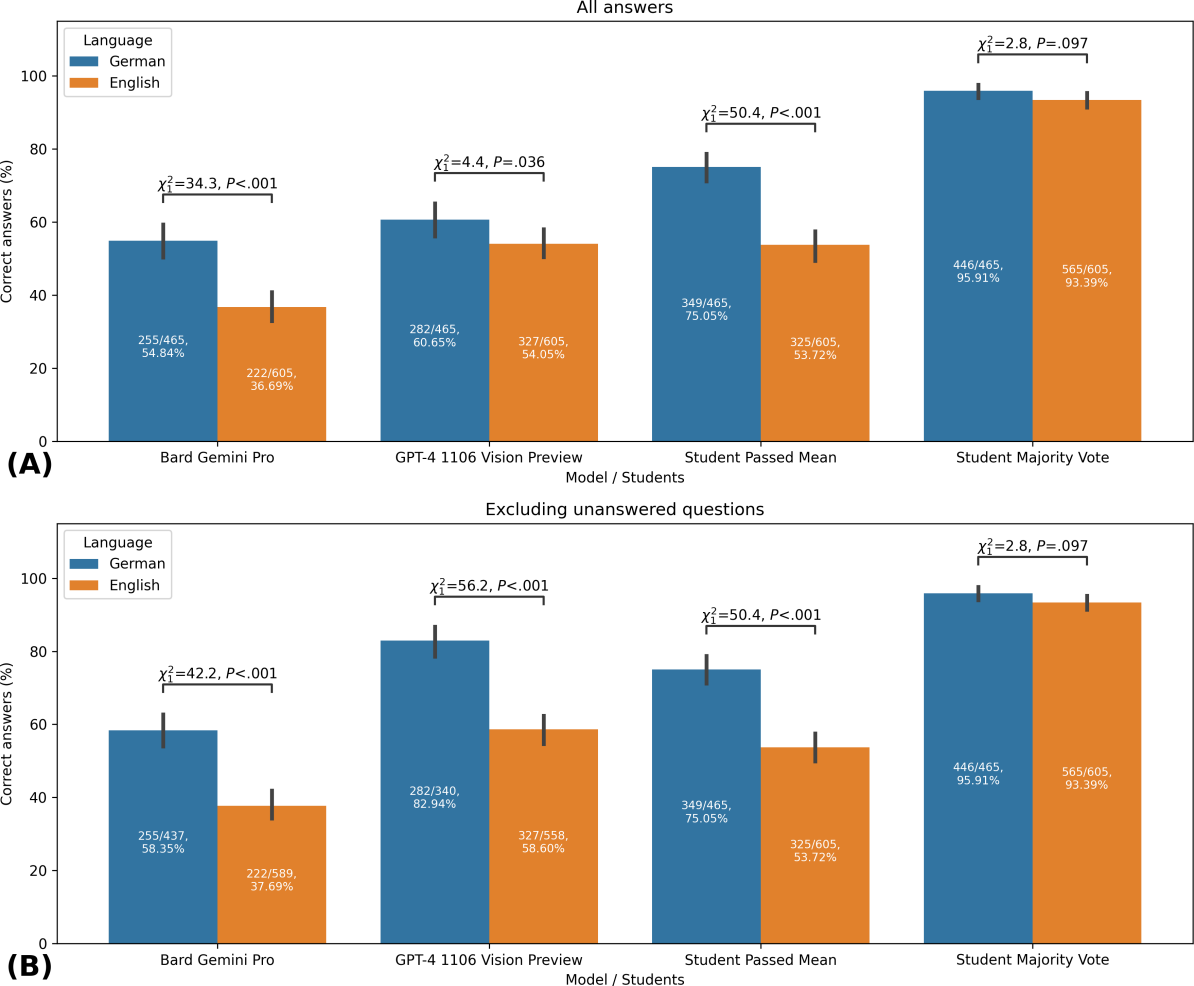
Performance comparison of AI models and students on medical visual question-answering tasks in English and German. AI: artificial intelligence.

The students also exhibited significant differences, achieving greater accuracy in German (349/465, 75.1%) over English (325/605, 53.7%; *χ*^2^₁=50.4, *P*<.001) when considering the mean score, a trend that was consistent in the subset of answered questions. The student majority vote maintained a high accuracy in both languages (German: 446/465, 95.9%, English: 565/605, 93.4%; *χ*^2^₁=2.8, *P*=.10), with no statistically significant difference between languages (Figure 3).

This figure presents the accuracy rates of Bard Gemini Pro, GPT-4 1106 Vision Preview, and medical students in answering image-based multiple-choice questions from medical licensing examinations. This study, conducted in 2024, analyzed 1070 questions (605 in English and 465 in German) from the AMBOSS learning platform. Panel A shows the results for all questions, while panel B displays results excluding unanswered questions. This study was conducted in 2023, comparing AI model performance against medical student performance data from March 21, 2023 (German questions), and June 16, 2023 (English questions). The bars represent the percentage of correct answers for each group, separated by language. Statistical significance was determined using chi-square tests to compare accuracy between German and English questions within each group. The figure illustrates language-specific performance differences and compares AI models’ capabilities with student performance.

### Bard Gemini Pro Prompt Safety Evaluation

Our analysis of Bard Gemini Pro’s content evaluation was conducted for both the German and English languages (Table 2). The results showed a low number of issues, which contrasts with the high number of unanswered questions for both models.

**Table 2.** Safety evaluation of medical visual question-answering prompts by Bard Gemini Pro [21].

Language and evaluation^a^		Sexually explicit	Hate speech	Harassment	Dangerous content
**German**					
	Low	1	2	2	0
	Negligible	436	435	435	437
**English**					
	Low	0	3	2	0
	Negligible	589	586	587	589
**Overall**					
	Low	1	5	4	0
	Negligible	1025	1021	1022	1026

a ”Negligible” indicates a negligible chance of unsafe content, while “low” suggests a low probability according to Google’s proprietary classification system.

This table presents the results of Bard Gemini Pro’s content safety evaluation for 1026 out of 1070 (96%) medical image-based multiple-choice questions. The evaluation categorizes potential safety concerns into four types: sexually explicit content, hate speech, harassment, and dangerous content. The results are shown separately for German (465 questions) and English (605 questions) prompts, as well as overall totals. “Negligible” indicates a negligible chance of unsafe content, while “low” suggests a low probability according to Google’s proprietary classification system [21]. The table demonstrates the AI model’s assessment of potential safety issues in medical educational content across two languages. Note that 44 (4%) questions were not evaluated due to internal errors in the AI system (“500 An internal error has occurred. Please retry or report in https://developers.generativeai.google/guide/troubleshooting”).

## Discussion

### Principal Findings

Our study shows that GPT-4 1106 Vision Preview and Bard Gemini Pro have potential for answering medical questions, with a better performance in German. Overall, our results show room for improvement and the obvious need for improved adaptability in multilingual contexts and a deeper understanding of their limitations. By comparing two LLMs such as GPT-4V and Gemini Pro, medical education can be significantly improved in several ways. With the performance of different LLMs compared, educators can determine which model performs best on specific tasks, such as interpreting medical images or answering complex medical questions. This helps in selecting the most appropriate model for specific training requirements. LLMs can be used to train medical students by providing immediate feedback on diagnostic exercises. Comparing models ensures that the chosen LLM provides the most accurate and helpful feedback, improving students’ diagnostic skills. In addition, comparing LLMs can highlight gaps and limitations in their performance, leading to future improvements and training methods. Understanding these differences also helps in developing strategies to effectively integrate AI tools into the medical curriculum to enhance both the teaching and learning experience.

Compared to the scarce existing literature on image analysis studies of LLMs, our results seem to significantly outperform previous results, for example, in the detection of melanoma [22]. Compared to other AIs, the results appear to be expandable [23-25]. As these were developed for specific questions in comparison to LLMs, the results are nevertheless solid, which shows the great potential of these programs for the future.

Reliable image analysis could thus be extensively used in medical education. Currently, there are considerations to use ChatGPT in designing curricula, preparing lecture materials, and examination preparation [26]. With the improved results of ChatGPT 4 Vision over GPT-4 in clinical queries, these programs can be further used in future teaching and training [27]. In our study, the collective majority vote of students significantly outperformed both AI models, illustrating the value of collective human intelligence and the existing limitations of the models under investigation. However, further improvements to these models could potentially outperform collective human performance.

While ChatGPT-4 has repeatedly demonstrated excellent performance in medical licensing examinations, there are currently no studies investigating Gemini Pro’s capabilities in this context [6,7,28]. In previous analyses, media-related questions involving graphs, pictures, or clinical image data, had to be excluded [29]. The ability to analyze images is critical in many medical specialties. The analysis of radiographic images in orthopedics and trauma surgery, understanding dermatological findings, and interpreting electrocardiograms in cardiology are just a few of the essential skills for physicians. Developing these skills takes time and practice. With advances in image analysis capabilities, these skills could be incorporated into the training of medical students and residents. If reliable, LLMs could potentially enhance training by explaining imaging findings. Our results show a promising start in this direction, yet further optimization is needed to avoid misdiagnosis. The incorporation of AI fundamentals, practical application, and the development of reflective skills are essential for future medical education. However, there is a risk that these programs could be used by nonmedical professionals, potentially exposing patients to misdiagnosis.

As with the LLM models, the students showed language-related differences in performance and achieved a higher mean accuracy in German than in English. This unexpected finding could be attributed to various factors, including potential differences in question complexity, the specificity of German medical terminology, or the quality of German medical data in the training sets. It is also possible that the models benefit from cross-lingual transfer learning or, paradoxically, may be overfitted to certain patterns in English medical texts. This disparity underscores the need for further investigation into the language-specific performance of multilingual AI models in specialized domains such as medicine, with future research controlling for question complexity and content across languages to isolate the effect of language on model performance. In contrast it was observed that linguistic discrepancies in security evaluations impacted performance; notably, GPT-4 1106 Vision Preview did not respond to 125/465 (26.9%) of German queries as opposed to 47/605 (7.8%) in English, suggesting overly strict moderation for non-English content. Thus, while a higher accuracy was achieved, there was also a greater proportion of queries not answered at all. There are multiple factors that could influence this, including the difficulty level of the questions, the quality of the images, and the formulation of the questions. The reasons for this must be further investigated in future analyses.

In addition to assessing the medical visual question-answering performance, our study also examined Bard Gemini Pro’s content evaluation for potential issues such as sexually explicit material, hate speech, harassment, and dangerous content. This analysis, conducted in both German and English languages, revealed a low incidence of such issues. Interestingly, this finding contrasts with the high number of unanswered questions observed in both models. Bard Gemini Pro showed only a small proportion of questions with problematic content, which shows that there is currently a transparency problem with the model. The questions do not appear to be problematic, but they are still not answered. It must be critically noted that there are no explanations as to why certain content is filtered and not answered. This limits both the function and the scientific usability, as it restricts the comparability of the analysis.

It also raises the question of why more German than English questions are filtered. One explanation could be that the systems overregulate in languages they are not trained in. However, the questions are not directly comparable. This requires further analysis of the extent to which the language and the given content have an influence on the answers to the questions.

Overall, our analysis suggests that the image analysis function has limitations despite relatively good results. The specific reasons for the inability to answer certain questions remain unclear. The meta-feedback from Bard Gemini Pro regarding safety categories is a crucial aspect that reflects the ongoing efforts to make these models safe and ethical, and this should be further elaborated.

### Limitations

Our study’s focus on German and English datasets limits its applicability to a global context, particularly in less common languages. In addition, our analysis was limited to specific versions of GPT-4 1106 Vision Preview and Bard Gemini Pro and did not include other models or iterations. Due to the significant number of unanswered questions, the true overall accuracy of the models can only be guessed within the given results. While these results are promising, they lack real-world clinical validation, which is crucial for drawing firm conclusions. Additionally, the English and German questions were not identical, introducing a discrepancy that makes the validity of the language comparison not fully accurate. Furthermore, the performance of these models may vary significantly in actual clinical settings, where diagnostic reasoning involves the integration of complex patient data. It is important to note that the English and German questions in our study were not identical, which introduces a potential confounding factor in our language comparison. This limitation means that differences in performance between languages could be due to variations in question difficulty or content rather than language effects alone. Future studies should consider using a set of equivalent questions translated into multiple languages to provide a more robust comparison of language-specific performance

### Conclusions

GPT-4 1106 Vision Preview and Bard Gemini Pro demonstrated potential in medical visual question-answering tasks, with GPT-4 outperforming Bard (609/1070, 56.9% vs 477/1070, 44.6% accuracy) and showing higher accuracy in German than English. Both models, however, fell short of the student collective majority vote (1011/1070, 94.5% accuracy), highlighting current limitations in AI performance for medical image interpretation. These findings suggest that while AI models show promise as educational tools, they require further optimization to enhance accuracy, language adaptability, and consistency before they can be reliably implemented in clinical settings.

## Supplementary material

10.2196/57592 Multimedia Appendix 1 Bard Gemini Pro and GPT-4 1106 Vision Preview model responses.

10.2196/57592 Multimedia Appendix 2 Model responses.

## Abbreviations

AI: artificial intelligence
LLM: large language model
