# Correction: Evaluating Bard Gemini Pro and GPT-4 Vision Against Student Performance in Medical Visual Question Answering: Comparative Case Study

**DOI:** 10.2196/71664

**Published:** 2025-02-11

**Authors:** Jonas Roos, Ron Martin, Robert Kaczmarczyk

**Affiliations:** 1 Department of Orthopedics and Trauma Surgery, University Hospital of Bonn, Venusberg-Campus 1, 53127, Bonn, Germany, 49 228-287-14170; 2 Department of Plastic and Hand Surgery, Burn Center, BG Clinic Bergmannstrost, Halle (Saale), Germany; 3 Department of Dermatology and Allergy, Technical University of Munich, Munich, Germany

In “Evaluating Bard Gemini Pro and GPT-4 Vision Against Student Performance in Medical Visual Question Answering: Comparative Case Study” (JMIR Form Res 2024;8:e57592) the authors noted two errors.

MultimediaAppendices 1  2 contained identical files. Therefore, the Multimedia Appendix 1 file has been changed to the correct supplementary inference code.

Additionally, the “query” column of Multimedia Appendix 2 has been removed.

The corrections will appear in the online version of the paper on the JMIR Publications website on February 11, 2025, together with the publication of this correction notice. Because this was made after submission to PubMed, PubMed Central, and other full-text repositories, the corrected article has also been resubmitted to those repositories.

## Supplementary material

10.2196/71664 Multimedia Appendix 1 Inference code.

10.2196/71664 Multimedia Appendix 2 Responses from the models.

